# New model to predict survival in advanced pancreatic ductal adenocarcinoma patients by measuring GGT and LDH levels and monocyte count

**DOI:** 10.3389/fonc.2024.1411096

**Published:** 2024-10-07

**Authors:** Rocío del Campo-Pedrosa, Alfonso Martín-Carnicero, Ana González-Marcos, Alfredo Martínez

**Affiliations:** ^1^ Data Department, Encore Lab S.L., Logroño, Spain; ^2^ Department of Mechanical Engineering, Universidad de La Rioja, Logroño, Spain; ^3^ Medical Oncology Department, Hospital San Pedro, Logroño, Spain; ^4^ Angiogenesis Group, Center for Biomedical Research of La Rioja (CIBIR), Logroño, Spain

**Keywords:** prognosis model, prognostic biomarkers, overall survival, gamma glutamyl transferase (GGT), lactate dehydrogenase (LDH), advanced and metastatic pancreatic cancer, pancreatic ductal adenocarcinoma (PDAC)

## Abstract

**Introduction:**

Pancreatic ductal adenocarcinoma (PDAC) is a lethal cancer with a poor survival outcome. Predicting patient survival allows physicians to tailor treatments to specific individuals. Thus, a simple and cost-effective prognosis model is sorely needed.

**Methods:**

This retrospective study assesses the prognostic value of blood biomarkers in advanced and metastatic PDAC patients (n=96) from Spain. Cut-off points for hematological parameters were calculated and correlated with overall survival (OS) using Kaplan-Meier, log-rank test, robust Cox proportional hazards and logistic regressions.

**Results:**

In univariate analysis, individuals with low levels of GGT, LDH, ALP, leukocyte-, neutrophil- and monocyte counts showed significantly longer survival than patients with higher levels. In multivariate analysis, lower levels of GGT (HR (95%CI), 2.734 (1.223-6.111); p=0.014), LDH (HR (95%CI), 1.876 (1.035-3.400); p=0.038) and monocyte count (HR (95%CI), 1.657 (1.095-2.506); p = 0.017) remained significantly beneficial. In consequence, we propose a prognostic model based on logistic regression (AUC=0.741) of these three biomarkers as a pioneer tool to estimate OS in PDAC.

**Conclusion:**

This study has demonstrated that the joint use of GGT (<92.00), LDH (<220.00) and monocyte count (<800) are independent positive prognostic factors in PDAC that can predict one-year survival in a novel prognostic logistic model.

## 1 Introduction

Pancreatic ductal adenocarcinoma (PDAC) is one of the most lethal malignancies (1) and is associated with a devastating prognosis and a 5-year survival rate of only 12.8% (2). The poor clinical outcomes are a consequence of being a mostly asymptomatic condition with nonspecific symptoms during its initial phase (3). As a consequence, PDAC patients are usually diagnosed either at an advanced stage (30–35%) or when already presenting metastatic disease (50–55%) (4), resulting in limited treatment options and an unfavorable outcome.

According to the National Center for Health Statistics, in the United States around fifty thousand people were diagnosed and died from pancreatic cancer in 2023 (1), meanwhile the latest worldwide statistics indicate that approximately half a million people died from this disease in 2020 (5, 6). PDAC incidence varies across countries, being most prevalent in Europe and North America, while being less frequent in Africa and Central Asia. Worldwide, PDAC incidence continues rising by 1.0% per year (1) and it is projected to soar by 1.55% per year in the period 2019-2039 (7) and to become the second-leading cause of cancer-related mortality by 2030 (4, 8).

In Spain, according to the latest estimates for 2023 (9), pancreatic cancer ranks 8th in incidence both in men and women, with 9,280 cases per year (4,770 cases in men and 4,510 in women). PDAC is among cancers that cause the most deaths in Spain and both, its incidence and mortality, are increasing. Net survival is less than a year for both men and women.

Current treatment approaches for PDAC are determined by cancer’s stage and location, as well as by the patient’s overall health (10). Early-stage diagnosis may lead to surgery, possibly with adjuvant or neoadjuvant chemotherapy. In the event of a relapse, or for those who are not eligible for surgery, patients receive chemotherapy regimens such as FOLFIRINOX or gemcitabine with capecitabine, cisplatin, or nab-paclitaxel (11). Notwithstanding, the sole curative approach is complete surgical resection (3), whenever feasible. The resection rates and use of adjuvant chemotherapy have increased in the last two decades, although still the majority of patients only received supportive care (12).

Blood biomarkers could be used as prognostic variables. So far, several studies have analyzed the relationship between minimally-invasive biomarkers and survival in different cancers. These works have identified significant biomarkers for prognosis, such as blood cell ratios in PDAC, non-small-cell lung cancer, and colorectal cancer, among others (13); lactate dehydrogenase (LDH) and alkaline phosphatase (ALP) in breast cancer (14), or serum uric acid (SUA) ratio and gamma glutamyl transferase (GGT) in rectal cancer (15). Regarding pancreatic cancer, different biomarkers related to oncogenic mutations (KRAS, CDKN2A, TP53 or SMAD4) or blood biomarkers (CA 125, CA 19.9, CEA, ADH, MIC-1, TIMP1, etc.) have been proposed (3). Furthermore, other hematological indices (blood cell ratios, GGT, LDH, ALP, etc.) have drawn the attention to their prognostic capability in pancreatic cancer (16–23).

The aim of this study is to analyze the potential of different blood biomarkers as prognostic instruments in locally advanced and metastatic pancreatic cancer to generate a non-invasive and cost-effective prognosis model. In particular, there is a scarcity of studies addressing the impact of neat GGT serum levels in advanced or metastatic PDAC patients (19, 24, 25) and none have examined its combined effect with LDH, despite both, GGT (15, 26–29) and LDH (14, 30–33), having a potential prognosis value in various cancer contexts. Hence, the present study aims to examine how these biomarkers are related and determine their collective potential as predictors of survival along any systemic inflammation parameter which may imply poor outcomes in different cancers, including PDAC (20). To achieve this, clinical data were correlated with overall survival (OS) in a cohort of PDAC patients who had been treated at a local hospital in La Rioja (Spain).

## 2 Materials and methods

### 2.1 Patients

PDAC patients were retrospectively identified at the Medical Oncology department registry at San Pedro University Hospital in Logroño (La Rioja, Spain). All patients were older than 18 years and died from metastatic pancreatic cancer. Patients were diagnosed and treated at the San Pedro Hospital in the period between 2010 and 2021.

Initially, 143 patients were pre-selected, but due to missing blood sample values, the study was conducted with 96 patients. This cohort included both patients who presented synchronous (at diagnosis) or metachronous (during the evolution of their disease) metastases. The study protocol was approved by the Medical Research Ethics Committee of La Rioja (CEICLAR, protocol number 260).

### 2.2 Treatment protocols

All patients were treated with one, two or more lines of chemotherapy according with standard protocols, including FOLFIRINOX, Gemcitabine plus Nab-paclitaxel, FOLFOX-6, FOLFIRI, Gemcitabine plus Capecitabine and Gemcitabine.

### 2.3 Study variables

Clinical and biochemical data included in the analysis are sex, age at diagnosis, presence of metastasis at diagnosis, presence of liver metastasis, Eastern Cooperative Oncology Group (ECOG) performance status, and blood variables: glucose, bilirubin, alkaline phosphatase (ALP), gamma glutamyl transferase (GGT), total lactate dehydrogenase (LDH), hemoglobin (Hb), number of leukocytes, neutrophiles, lymphocytes, monocytes, and platelets, neutrophile-lymphocyte ratio (NLR), lymphocyte-monocyte ratio (LMR) and platelet-lymphocyte ratio (PLR). Clinical stage was established according to the 8th edition of the AJCC (cTNM) and confirmed by body CT (34).

In addition, progression-free survival (PFS) was calculated, defined as the time interval between the start of chemotherapy and the date of relapse, and overall survival (OS) as the time elapsed from the date of diagnosis to the day of exitus.

### 2.4 Statistical analysis

Patients were divided into two groups depending on their OS at one-year post-diagnosis. With the entire sample regrouped according to survival, and after verifying that the assumptions of normality were not satisfied, the U Mann-Whitney test was used to evaluate differences in numerical variables and the chi-square test for binary variables. For blood variables, the Youden Index methodology was performed to find the optimal cut-off (35).

Survival analysis was carried out using the Kaplan-Meier curve and the log-rank test. This methodology was used to assess whether the cut-off points were statistically significant in terms of OS.

Finally, blood variables grouped by the cut-off, alongside the rest of characteristics, were examined using univariate and multivariate Cox proportional hazards regression. Since outliers were identified, the robust Cox method proposed by Bednarski (36) was employed. These outliers were evaluated through a consensus approach that integrates multiple outlier detection techniques based on residuals, concordance-index and censored quantile regression methodologies, obtaining a unified consensus ranking achieved via the calculation of the rank product (RP) (37). Specifically, martingale residuals, deviance residuals, bootstrap hypothesis test, dual bootstraps hypothesis testing, as well as residual and score algorithms were utilized as outlier detection methods. In addition, a multivariate logistic regression was designed and adjusted to an adequate proportion of events per variable (EPV) to ensure robust performance (38, 39). Both multivariate models, presented with the current cohort of patients, were validated using bootstrap techniques.

All statistical analyses were performed using R version 4.2.2 software (40). Test results were deemed statistically significant when p-value was lower than 0.05. In the case of the RP test, to regulate the false discovery rate (type I errors) in multiple testing, the q-value was employed as the metric to ascertain the False Discovery Rate (FDR). Thus, outliers were regarded as significant when the q-value was lower than 0.05.

## 3 Results

### 3.1 Patients characteristics

The sample is comprised of 96 individuals, 52 of whom were men (54.2%), and 44 were women (45.8%), with a median (Q1-Q3) age of 67 (60–73) years (**Table 1**). According to their ECOG performance status, 42 individuals were diagnosed with ECOG 0 (43.8%), 42 with ECOG 1 (43.8%) and the rest with ECOG 2 (12.5%). Of this set of patients, at diagnosis 63 (65.6%) presented synchronous metastases (stage IV), while the rest developed them metachronously (stage III). By the end of the study, 50 patients (52.1%) had developed liver metastases (**Table 1**).

**Table 1:** Patients’ characteristics and treatments.

Characteristic	Patients
Age at diagnosis (mean ± sd)	66.08 ± 9.62
Female: Male (n)	44:52
Metastases, n (%)	63 (65.6%)
Liver Metastases, n (%)	50 (52.1%)
ECOG0:ECOG1:ECOG2 (n)	42:42:12
StageIV: StageIII: StageIIB-IIA-IB (n)	63:21:12
Perioperative Treatment, n (%)	5 (5.2%)
Chemotherapy Treatment	
• No Line, n (%)	1 (1.0%)
• First-line, n (%)	95 (99.0%)
• Second-line or more, n (%)	52 (54.2%)
Chemotherapy first-line	
• FOLFIRINOX, n (%)	25 (26.0%)
• Gemcitabine+Capecitabine, n (%)	1 (1.0%)
• Gemcitabine+Nab-paclitaxel, n (%)	29 (30.2%)
• Gemcitabine, n (%)	38 (39.6%)
• FOLFOX-6, n (%)	2 (2.1%)

All patients received chemotherapy. Specifically, 95 (99.0%) individuals underwent first-line, of which 52 continued with at least second-line (54.2% of the total sample) and one of them received perioperative treatment (**Table 1**).

### 3.2 Survival

The median OS (Q1–Q3) was 9.18 (4.48-13.82) months with a mean of 9.95 months. The OS cut-off point was set at 12 months, resulting in 35 cases with survival rates of one year or more (36.5%), and 61 with less (63.5%).

For the individuals treated with first-line of chemotherapy (99.0%), the median PFS (Q1-Q3) was 3.84 (1.88-7.10) months, with a mean of 5.04 months. Specifically, cases with survival rates of less than a year presented a median PFS (Q1-Q3) of 2.60 (1.10-5.07) months whereas the others had a PFS of 8.47 (6.27-10.93) months.

### 3.3 Prognosis factors

In the univariate analysis in the cohort grouped by a one-year OS, statistically significant differences were obtained for the parameters “general metastasis” (Stage IV vs Stage III) but not in “liver metastasis” (**Table 2**). Regarding blood markers, the parameters LDH, neutrophile and monocyte count presented statistically significant differences between the survival groups (**Table 2**). GGT levels were non-significant but close to the threshold (p=0.071).

**Table 2:** Individuals’ characteristics and hematologic parameters comparing survival groups.

variable	Group OS≥12	Group OS< 12	p-value
Number of patients, n (%)	35 (36.5%)	61 (63.5%)	0.008**
Age at diagnosis (mean ± sd)	67.97 ± 7.33	65.00 ± 10.62	0.290
Female: Male (n)	17:18	27:34	0.845
Metastasis, n (%)	18 (18.8%)	45 (46.9%)	0.046*
Liver Metastasis, n (%)	15 (15.6%)	35 (36.5%)	0.247
ECOG0:ECOG1:ECOG2 (n)	18:14:3	24:28:9	0.449
Glucose	112.00 ± 59.50	114.00 ± 65.5	0.584
Bilirubin	0.70 ± 4.45	0.70 ± 2.45	0.948
ALP (mean ± sd)	124.00 ± 267.03	177.00 ± 266.09	0.146
GGT (mean ± sd)	103.00 ± 452.57	221.00 ± 513.13	0.071
LDH (mean ± sd)	194.00 ± 141.81	261.00 ± 218.73	0.037*
Hb (mean ± sd)	12.90 ± 1.68	13.00 ± 2.09	0.570
Leukocytes (mean ± sd)	7,400 ± 2,740	8,200 ± 2,885	0.051
Neutrophiles (mean ± sd)	4,200 ± 2,846	5,600 ± 2,630	0.047*
Lymphocytes (mean ± sd)	1,500 ± 752	1750 ± 901	0.740
Monocytes (mean ± sd)	600 ± 217	800 ± 1,211	0.042*
Platelets (mean ± sd)	219,000± 114,927	246,000± 141,646	1.000
NLR (mean ± sd)	2.53 ± 3.67	2.95 ± 3.39	0.188
LMR (mean ± sd)	2.80 ± 1.68	2.38 ± 6.72	0.194
PLR (mean ± sd)	144.00 ± 95.15	133.10 ± 102.84	0.667

Asterisks represent statistically significant differences. *: p<0.05; **: p<0.01.

The optimal cut-off point was found for each blood parameter through the Youden index (35) so that these variables could be used to determine a model suitable for clinical practice (**Table 3**). Although the Youden Index test showed moderate discriminative ability (AUC<0.7), the cut-off points proved effective in distinguishing OS. Kaplan-Meier analysis shows that patient’s groups divided by the optimal cut-off of ALP, GGT, LDH, and numbers of leukocytes, neutrophiles and monocytes were statistically significant (**Tables 3**, **4**).

**Table 3:** Youden Index cut-off points for hematologic parameters.

variable	normal range	optimal cut-off	YI (Youden Index)	AUC	p-value (log-rank)
Glucose	70-100 (mg/dL)	122.00	0.1855	0.5337	0.052
Bilirubin	0.0-1.2 (mg/dL)	0.60	0.0965	0.4960	0.491
ALP	40-129 (U/L)	102.00	0.2361	0.5895	0.004**
GGT	10-71 (U/L)	92.00	0.2562	0.6112	0.031*
LDH	120-150 (U/L)	220.00	0.3457	0.6281	0.002**
Hb	12-16 (g/dL)	12.30	0.1171	0.5349	0.872
Leukocytes	4,500-11,000	8,000	0.2881	0.6199	0.006**
Neutrophiles	1,900-8,000	6,000	0.2304	0.6220	0.024*
Lymphocytes	4,000-11,000	2,500	0.0810	0.4796	0.916
Monocytes	200-1,000	800	0.2675	0.6244	0.016*
Platelets	15,000-400,000	326,000	0.0642	0.5000	0.632
NLR	–	1.95	0.2197	0.5810	0.072
LMR	–	3.00	0.1742	0.5799	0.108
PLR	–	64.49	0.0694	0.4735	0.231

Asterisks represent statistically significant differences. *: p<0.05; **: p<0.01.

**Table 4:** Kaplan-Meier and log-rank values for statistically significant hematologic parameters grouped by the optimal cut-off.

Variable	median survival (95%CI) (months)				
	<cut-off point	≥cut-off point	N	χ2	p-value
ALP	12.67 (10.17-17.80)	7.37 (5.77-10.10)	24/72	8.4	0.004**
GGT	12.30 (8.97-15.30)	7.47 (6.07-10.10)	31/65	4.7	0.031*
LDH	12.75 (11.40-14.57)	6.42 (4.50-8.93)	42/54	9.8	0.002**
Leukocytes	11.80 (9.50-13.40)	6.50 (4.93-9.63)	51/45	7.5	0.006**
Neutrophiles	11.15 (8.97-12.80)	6.42 (4.40-9.40)	60/36	5.1	0.024*
Monocytes	11.50 (9.50-12.90)	6.40 (5.23-8.93)	55/41	5.8	0.016*

Asterisks represent statistically significant differences. *: p<0.05; ** : p<0.01.

**Figure 1:**
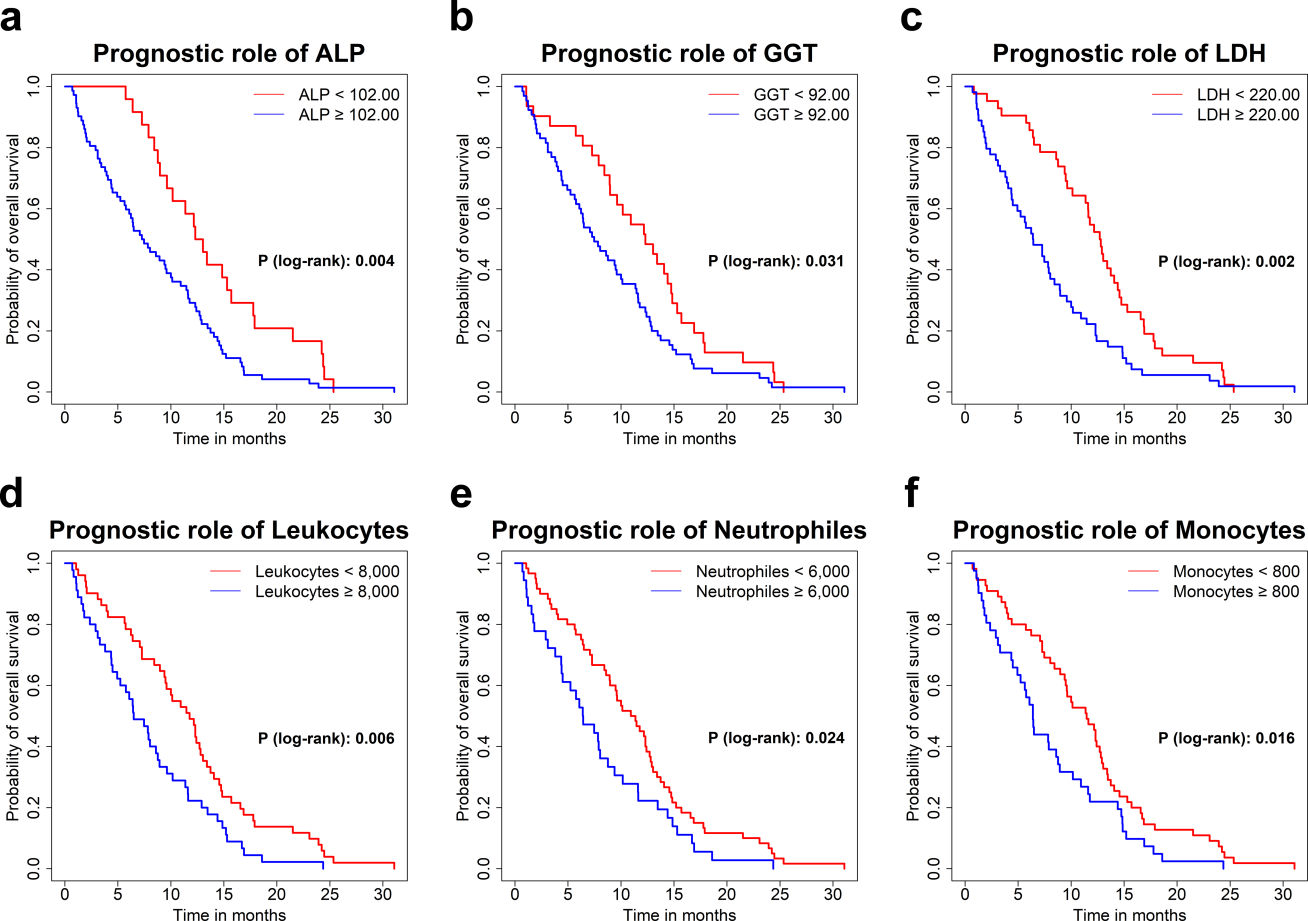
Kaplan-Meier survival curves for ALP **(A)**, GGT **(B)**, LDH **(C)**, Leukocyte count **(D)**, Neutrophile count **(E)** and Monocyte count **(F)** by optimal cut-off.

Interestingly, only the cut-off points of GGT<92.00 and LDH<220.00 were established outside of the normal clinical range (**Table 3**). Those individuals whose values on GGT and LDH were below the cut-off point (**Table 4**) presented a better prognosis than the ones who were above; specifically, they presented an OS increase by 39.3%, and 49.6%, respectively (**Figures 1B, C**). For their part, ALP<102.00 (**Figure 1A**), number of leukocytes<8,000, neutrophiles<6,000 and monocytes<800 (**Figures 1D–F**) were also statistically significant, resulting in patients with low levels increasing their survival by 41.8%, 44.9%, 42.4% and 44.3%, respectively (**Table 4**).

**Figure 2:**
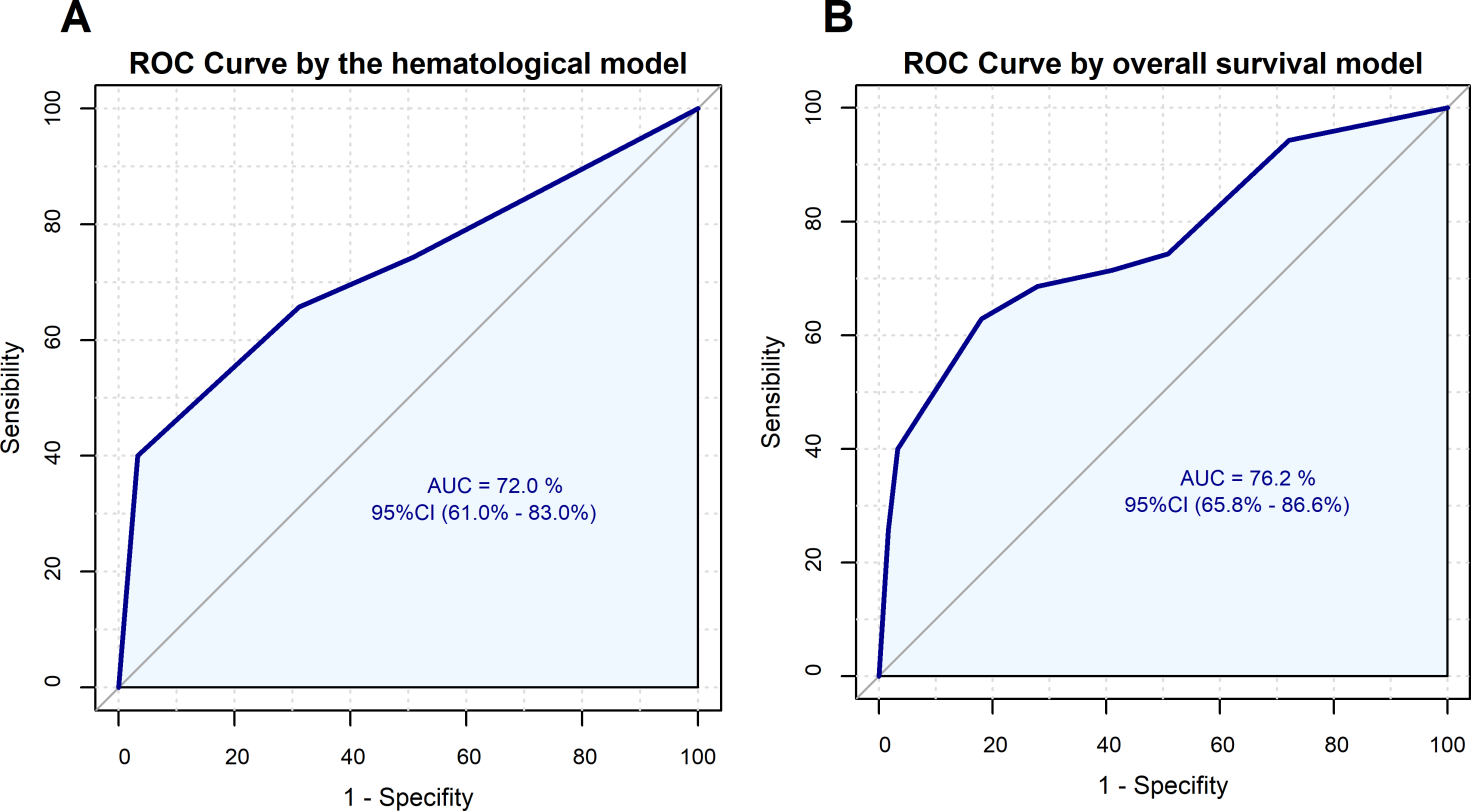
ROC curves for the logistic regression models. **(A)** ROC curve for one-year OS by GGT and LDH. **(B)** ROC curve for one-year OS by GGT, LDH and monocyte count.

Although several blood parameters showed prognostic power, we wanted to propose a joint prognostic model for PDAC patients. First, we prepared a multivariate analysis by implementing a logistic model to assess the ability of neat GGT and LDH values as predictors. This model generated a ROC curve with an AUC=0.720 (**Figure 2A**), and showed that GGT≥92.00 (95% CI 0.127-0.843; p = 0.021) and LDH≥220.00 (95% CI 0.097-0.604; p = 0.002) maintained their statistical significance (**Table 5**). The model presented an EPV=17.5, which is well above the proposed minimum of EPV≥10 (38). Since the model presented an EPV close to 20, a bootstrap-corrected approach and an independent validation subset should present negligible differences (39). Accordingly, this model was internally validated using bootstrapping (with 1,000 iterations). The result was a corrected AUC of 0.710. After verifying all the models, LDH was found statistically significant in 87.6% of the models and GGT in 64.7%.

**Table 5:** Logistic regression considering hematologic parameters GGT and LDH.

variables	Coef	Std Error	Z	Exp B	Lower CI_95	Upper CI_95	p-value
(Intercept)	0.903	0.453	1.994	2.467	1.016	5.993	0.046*
GGT≥92.00	-1.117	0.483	-2.314	0.327	0.127	0.843	0.021*
LDH≥220.00	-1.417	0.466	-3.043	0.242	0.097	0.604	0.002**

Asterisks represent statistically significant differences. *: p<0.05; **: p<0.01.

Next, we decided to train a model with a higher accuracy. Thus, we combined other significant biomarkers along GGT and LDH to increase the prognosis value. Under the criterion of achieving a high robustness based on EPV assumptions, where it has been demonstrated that using the recommended EPV=10 produced negligible bias in the 3- and 5-predictor models (38), we decided to adequate our proposed prognosis model to 3 predictor variables resulting in a model of EPV=11.75, slightly higher than the recommendation for robustness. Thus, we finally adjusted a logistic model including GGT and LDH levels and monocyte count (**Table 6**). As a result, a ROC curve with AUC=0.762 was obtained (**Figure 2B**). We found a model where GGT (95% CI 0.112-0.806; p = 0.017), LDH (95% CI 0.099-0.653; p = 0.004), and monocyte count (95% CI 0.112-0.829; p = 0.020) remained statistically significant. This model was internally validated using bootstrapping (with 1,000 iterations); the result was a corrected AUC of 0.741. In other words, the model is capable of distinguishing between groups to successfully predict one year survival. After contrasting all the validation models, monocyte count was found statistically significant in 65.0% of the models, GGT in 66.9% and LDH in 82.2%. Finally, we calculated the power (1-β) for the model, which presented a value of 0.97. This high power, well-above the usual required setting of 0.80, indicated that our model is able to detect the effect with no need of additional samples to be trained.

**Table 6:** Logistic regression for overall survival considering GGT, LDH and monocyte count.

variables	Coef	Std Error	Z	Exp B	Lower CI_95	Upper CI_95	p-value
(Intercept)	1.388	0.521	2.662	4.005	1.442	11.125	0.008**
GGT≥92.00	-1.204	0.504	-2.389	0.300	0.112	0.806	0.017*
LDH≥220.00	-1.370	0.481	-2.848	0.254	0.099	0.653	0.004**
Monocytes≥800	-1.186	0.510	-2.327	0.305	0.112	0.829	0.020*

Asterisks represent statistically significant differences. *: p<0.05; **: p<0.01.

Furthermore, these results were corroborated through the univariate and multivariate Proportional Hazards Model Cox regression. For the univariate analysis, patients with lower levels of the blood biomarkers presented better OS than patients with ALP≥102.00 U/L (HR (95%CI), 1.991 (1.239-3.201); p = 0.004), GGT≥92.00 U/L (HR (95%CI), 1.607 (1.041-2.48); p = 0.032), LDH≥220.00 U/L (HR (95%CI), 1.916 (1.269-2.892); p <0.002), number of leukocytes≥8,000 (HR (95%CI), 1.763 (1.166-2.665); p = 0.007), number of neutrophiles≥6,000 (HR (95%CI), 1.612 (1.059-2.454); p = 0.026) and number of monocytes≥800 (HR (95%CI), 1.657 (1.095-2.506); p = 0.017) (**Table 7**). For the multivariate analysis, a total of 6 cases were identified as outliers (q<0.05). Specifically, outlier diagnosis revealed that the event (death) occurred later than expected in all these cases, although we could not identify any reason for their longer survival and theses cases should be analyzed in more detail. Therefore, we employed a robust Cox model (36) to mitigate the impact of these outliers. This multivariate model (**Table 7**) included the high-risk characteristic of suffering from metastasis (HR (95%CI), 1.388 (0.751-1.827); p = 0.280), ECOG 1 (HR (95%CI), 1.278 (0.721-2.266); p = 0.400), ECOG2 status (HR (95%CI), 3.332 (1.049-10.583); p = 0.041), as well as the biomarkers studied in logistic regression, which still prevailed as a survival predictive value. Patients with GGT≥92.00 U/L (HR (95%CI), 2.734 (1.223-6.111); p = 0.014), LDH≥220.00 U/L (HR (95%CI), 1.876 (1.035-3.400); p = 0.038) and number of monocytes≥800 (HR (95%CI), 1.912 (1.085-3.369); p = 0.025) had worse outcome. The Cox regression model presented a concordance (C-index) of 0.702. Performing bootstrapping (n=1,000), the corrected concordance was 0.676 and after inspecting all the models, we found GGT and LDH statistically significant in 67.7% of the models, ECOG1 in 11.1%, ECOG2 in 54.4%, monocyte counts in 49.9% and metastases in 15.3% of them.

**Table 7:** Univariate and robust multivariate Cox analyses with sampling weights at different variables for one-year OS.

	univariate				multivariate			
	beta	Wald	HR (95% CI)	p-value	beta	Wald	HR (95% CI)	p-value
Age at diagnosis	-0.014	-1.172	0.986 (0.963-1.009)	0.241				
Sex	-0.050	-0.238	0.952 (0.633-1.431)	0.812				
Metastasis	0.254	1.161	1.289 (0.840-1.978)	0.246	0.328	1.167	1.388 (0.751-1.827)	0.280
Liver Metastasis	0.321	1.553	1.378 (0.919-2.065)	0.120				
ECOG 0	reference				reference			
ECOG 1	0.159	0.724	1.173 (0.762-1.805)	0.469	0.246	0.707	1.278 (0.721-2.266)	0.400
ECOG 2	0.850	2.550	2.339 (1.217-4.494)	0.011*	1.204	4.168	3.332 (1.049-10.583)	0.041*
ALP≥102.00 U/L	0.689	2.843	1.991 (1.239-3.201)	0.004**				
GGT≥92.00 U/L	0.474	2.140	1.607 (1.041-2.480)	0.032*	1.006	6.003	2.734 (1.223-6.111)	0.014*
LDH≥220.00 U/L	0.65	3.093	1.916 (1.269-2.892)	0.002**	0.629	4.299	1.876 (1.035-3.400)	0.038*
Leukocytes≥8000 mg/mL	0.567	2.691	1.763 (1.166-2.665)	0.007**				
Neutrophiles≥6000 mg/mL	0.477	2.227	1.612 (1.059-2.454)	0.026*				
Monocytes≥800 mg/mL	0.505	2.389	1.657 (1.095-2.506)	0.017*	0.648	5.033	1.912 (1.085-3.369)	0.025*
NLR≥1.95	0.418	1.781	1.519 (0.959-2.406)	0.075				
LMR≥3.00	-0.340	-1.598	0.712 (0.469-1.080)	0.110				
PLR≥64.49	0.548	1.184	1.730 (0.698-4.286)	0.236				

Asterisks represent statistically significant differences. *: p<0.05; **: p<0.01.

## 4 Discussion

In this study, we assessed the prognostic value of different blood parameters to propose a useful prognosis survival model. Among these parameters, we found that GGT and LDH levels are very informative on predicting the fate of advanced and metastatic PDAC individuals. The combined elevation of GGT and LDH levels, which is deleterious for the patients, might reflect both enhanced oxidative stress (indicated by GGT activity related to glutathione metabolism) and altered energy metabolism (indicated by LDH activity related to glycolysis) (41). This dual alteration can contribute to the aggressive behavior of tumors and the progression of liver diseases by promoting cancer cell survival, proliferation, and resistance to apoptosis. This is the first time that this combination is analyzed in a PDAC population.

Specifically, after searching for the optimal cut-off point, we demonstrated that GGT and LDH have a joint prognostic value by means of a logistic regression model and a robust multivariate Cox model. Finally, we improved the basic logistic model by adding monocyte count as a predictor, resulting in a novel prognostic model with a corrected AUC of 74.1%.

Lactate dehydrogenase (LDH), a metabolic enzyme involved in glycolysis, is regulated by the PI3K/Akt/mTOR pathways, the MYC oncogenic transcription factor, tumor hypoxia, and necrosis (17). Thus, LDH, closely related with the biological behavior of tumors, can predict the prognosis of tumor patients (41). In fact, high levels of LDH have been demonstrated as a poor prognosis factor for several cancers, including renal cell carcinoma, nasopharyngeal carcinoma, sarcoma, melanoma, prostate cancer, colorectal cancer and lung cancer (33).

In the PDAC context, LDH has been widely assessed for both advanced and early-stage patients. For instance, Xiao et al. found that an LDH level higher than 250U/L was associated with an increased risk of death in early-stage patients who underwent curative surgery. They also found a dose-response relationship between LDH levels and OS (42). In studies of patients who underwent palliative chemotherapy or radiochemotherapy, LDH levels were found to be significant in univariate analysis, with LDH >250 U/L implying a higher risk for both OS and PFS. However, this significance did not persist in multivariate analyses (43). During palliative second-line treatment, LDH levels were not found to be significant (44).

Focusing in advanced and metastatic pancreatic cancer, Xiao et al. assessed LDH levels comparing patients who received subsequent chemotherapy versus those who did not. A higher LDH level before treatment (≥250 U/L) was linked to an increased risk of death in patients receiving chemotherapy, along with age, sex, and albumin levels, in a multivariate analysis. However, for patients who did not undergo chemotherapy, the risk increase was not statistically significant (45). Several studies evaluated LDH as a biomarker in cohorts treated with different first-line regimens: Sorafenib in combination with gemcitabine vs. gemcitabine (23), gemcitabine-based chemotherapy (17, 22), and anti-PD-1 treatment (16). Some of these studies performed only univariate analyses where higher LDH levels always implied shorter survival, with a cut-off at 225 (U/L) (23) or at 475 U/L (22). Among the publications that performed multivariate Cox regression, Yu et al. identified LDH≥185 U/L as an independent prognostic predictor of OS in the training cohort whereas in the validation cohort only a statistical trend was found (17). Qiu et al. demonstrated that LDH<=265 U/L had a significant better outcome for both PFS and OS (16). Although these last two studies included other blood biomarkers in their multivariate analysis, such as NLR, LMR, PLR or CA19.9, neither of them considered the interaction with GGT or with the number of white blood cells. Our LDH cut-off was 220 U/L, which is close to the other published cut-off values.

Gamma-glutamyltransferase (GGT) is an enzyme crucial in maintaining the body’s redox balance by cleaving the gamma-glutamyl residue from glutathione (GSH), a significant antioxidant. Most diseases are associated with oxidative stress, coursing with increased GGT values. An elevated GGT activity is linked to liver and pancreatic abnormalities, due to its important role in maintaining homeostasis and responding to oxidative environments, such as inflammation following tissue damage (46). High GGT levels have been related to an elevated risk of multiples cancers (47), including pancreatic cancer (48). Interestingly, the levels of GGT have shown a prognostic role for survival in several cancers, including gastric cancer (26), breast cancer (29), intrahepatic cholangiocarcinoma (27) and ovarian cancer (28).

To the best of our knowledge, neat GGT levels are rarely analyzed in PDAC. In early-stage patients, they have been studied mostly in combination with other blood parameters, as a ratio. For instance, GGT levels were part of significant prognostic ratios in combination with either CA19-9 (18, 49), lymphocyte count (50) or albumin (51).

Nevertheless, a few examples of the use of GGT serum levels in advanced and metastatic PDAC can be found in the literature. Engelken et al. showed that GGT≥165 U/L predicted a statistically significant poorer survival in a multivariate analysis, along with the absence of therapeutic intervention and leukocytosis in a cohort with unresectable pancreatic cancer (24). Xiao et al. described that having serum GGT ≥ 48 U/L resulted in an increased risk of mortality in multivariate Cox hazards regression along with fasting plasma glucose (19). A more recent work has evaluated pretreatment GGT levels in a metastatic PDAC cohort who underwent first line nab-paclitaxel/gemcitabine treatment. The univariate analysis showed that GGT>45.5 U/L presented poor prognosis in all patients, as well as in individuals with liver metastasis (25). Despite having shown relevance for elevated GGT levels, none of these studies included specific white blood cell counts or LDH levels in their analyses. In addition, our cohort does not contain patients who underwent surgical or palliative treatment, as is the case in the previous references, which could introduce confounding information. Therefore, our analysis provides a novel approach in the impact of this biomarker in predicting OS for advanced and metastatic PDAC patients.

Systemic inflammation is considered a significant indicator of poor prognosis for many types of cancers, including PDAC, and it is increasingly recognized as a critical aspect of cancer. It negatively affects various cancer processes including tumor growth, survival, spread, blood vessel formation, and treatment effectiveness (20). In fact, in our univariate analysis, high levels of monocytes, neutrophiles and leukocytes were associated to poor survival; and monocyte count persisted as a significant factor in the multivariate analysis.

In terms of advanced and metastatic PDAC, monocytes are normally included in analyses as part of inflammatory ratios (LMR) (21, 52), and rarely considered as an independent predictor (20). Since there is a clear relevance of this white blood cell in cancer evolution, we included its effect in the multivariate analysis, resulting in an improved logistic model for OS prediction. Monocyte count was found significant in both multivariate Cox regression and logistic regression, agreeing with the current bibliography on the close relationship of this blood cell type and PDAC (53, 54).

Despite GGT and LDH having been shown to be potential biomarkers in the cancer context, their joint effect has never been studied in PDAC patients. Elevated levels of these two biomarkers are closely related with tumor formation and recurrence, affecting tumor initiation and progression (41). Furthermore, they were predictive of low survival through a logistic model. To enhance their predictive ability, we added a well-known poor prognosis factor, namely monocyte count ≥800. As a result, we propose a logistic model, where the predictor variables are statistically significant, capable to predict one-year survival with a corrected performance AUC of 74.1%. Despite the limited sample size of our cohort, we have conscientiously conducted our analyses with methodologies that have been widely recognized as robust for similar conditions. Thus, the logistic regression models were adjusted well above the minimum of EPV≥10 (38) and validated by bootstrap techniques (n=1,000).

Currently, no recognized predictive model is in use in the clinical practice for OS in advanced pancreatic cancer patients. Nevertheless, there are other robust models to predict recurrence after surgery (55) and radical resection (56) in PDAC individuals. Most current models are mainly nomograms based on a few risk characteristics such as age, stage, tumor size, etc. (57) that might not capture the complexity of cancer risk, leading to overestimation or underestimation (58). The inclusion of blood biomarkers may help in improving these models. For instance, Goldstein et al. included quantitative values of neutrophil-lymphocyte ratio and albumin levels, and although the model achieved a low predictive power (C-Index of 0.67), it became a more complex model. Nomograms, although widely used in oncology, require a precise interpretation and imply a risk of incorrect patient prognoses and treatment decisions (59). In consequence, our logistic model, based on non-high-risk binary variables (GGT, LDH and monocyte count), represents a novel approach to estimate overall survival in the PDAC population.

Given that combined high levels of GGT and LDH are risk factors associated with lower survival, we believe that effective LDH and GGT inhibitors may have therapeutic value for PDAC patients showing high levels of these markers. Although inhibitors of LDH are widely applied for treating various cancers (60), including PDAC (61), GGT inhibitors are usually toxic and require further development (62). In view of the predictive value of GGT, we believe it would be worth finding a suitable GGT inhibitor. Moreover, considering the joint risk effect of LDH and GGT levels, we believe the combined effects of these two potential inhibitors may have a positive impact on their efficacy. The strength of our study is the proposal of a non-invasive and easy-to-implement prognostic tool for advanced and metastatic PDAC patients (Stage III and IV), which is sorely needed in the field. Our proposed model is simple to use, to understand, to interpret and to apply to all patients diagnosed with PDAC since the diagnostic evaluation of all patients always involves regular blood test parameters. Thus, our data suggest that GGT and LDH levels, and the number of monocytes, measured before starting treatment are undemanding and cost-effective prognostic factors that can provide an estimation of OS, which in turn may be useful for individual therapy decisions. However, this study has some limitations. First, it has a limited sample size, which, despite the cautious statistical measures applied, might have affected the results, including a possibility of selection bias. Although the cohort was balanced in terms of sex and the presence of liver metastases, it was not balanced in terms of ECOG status. Notwithstanding, it is worth mentioning that after classifying the patients in terms of one-year survival, both groups presented a proportional distribution according to ECOG, sex and presence of liver metastases. These findings should be considered cautiously and validated in future studies with larger cohorts, preferably with patients from diverse health centers.

## 5 Conclusion

This study has confirmed the connection between high levels of GGT and LDH, and a high monocyte count, with a poor prognosis in pancreatic cancer. As a result, we have implemented a non-invasive, simple-to-use and cost-effective prognosis model based on a logistic regression. This pioneering model can predict one-year survival on PDAC patients with a correct AUC of 74.1%. This may constitute a useful tool for medical decision-making and personalized therapy.

## Data Availability

The raw data supporting the conclusions of this article will be made available by the authors, without undue reservation.
