# Corrigendum: New model to predict survival in advanced pancreatic ductal adenocarcinoma patients by measuring GGT and LDH levels and monocyte count

**DOI:** 10.3389/fonc.2024.1540165

**Published:** 2025-01-15

**Authors:** Rocío del Campo-Pedrosa, Alfonso Martín-Carnicero, Ana González-Marcos, Alfredo Martínez

**Affiliations:** ^1^ Data Department, Encore Lab S.L., Logroño, Spain; ^2^ Department of Mechanical Engineering, Universidad de La Rioja, Logroño, Spain; ^3^ Medical Oncology Department, Hospital San Pedro, Logroño, Spain; ^4^ Angiogenesis Group, Center for Biomedical Research of La Rioja (CIBIR), Logroño, Spain

**Keywords:** prognosis model, prognostic biomarkers, overall survival, gamma glutamyl transferase (GGT), lactate dehydrogenase (LDH), advanced and metastatic pancreatic cancer, pancreatic ductal adenocarcinoma (PDAC)

In the published article, there was an error in **Table 4** as published. Error was in the manual transcription of the numerical data of the Kaplan-Meier result. Specifically, the transcription error has occurred for the rows of ALP, GGT and LDH. The corrected **Table 4** and its caption “Kaplan-Meier and log-rank values for statistically significant hematologic parameters grouped by the optimal cut-off” appear below.

**Table 4:** Kaplan-Meier and log-rank values for statistically significant hematologic parameters grouped by the optimal cut-off.

Variable	median survival (95%CI) (months)				
	<cut-off point	≥cut-off point	N	χ2	p-value
ALP	12.67 (10.17-17.80)	7.37 (5.77-10.10)	24/72	8.4	0.004**
GGT	12.30 (8.97-15.30)	7.47 (6.07-10.10)	31/65	4.7	0.031*
LDH	12.75 (11.40-14.57)	6.42 (4.50-8.93)	42/54	9.8	0.002**
Leukocytes	11.80 (9.50-13.40)	6.50 (4.93-9.63)	51/45	7.5	0.006**
Neutrophiles	11.15 (8.97-12.80)	6.42 (4.40-9.40)	60/36	5.1	0.024*
Monocytes	11.50 (9.50-12.90)	6.40 (5.23-8.93)	55/41	5.8	0.016*

Asterisks represent statistically significant differences. *: p<0.05; **: p<0.01.

In the published article, there was an error. The error corresponds to the paragraph in which numerical references are made to the **Table 4** with the numerical transcription error.

A correction has been made to **Results**, *Prognosis*, Paragraph 3. This sentence previously stated:

“Those individuals whose values on GGT and LDH were below the cut-off point (**Table 4**) presented a better prognosis than the ones who were above; specifically, they presented an OS increase by 44.8%, and 74.6%, respectively (Figures 1B, C). For their part, ALP<102.00 (Figure 1A), number of leukocytes<8,000, neutrophiles<6,000 and monocytes<800 (Figures 1D–F) were also statistically significant, resulting in patients with low levels increasing their survival by 49.7%, 44.9%, 42.5% and 44.4%, respectively (**Table 4**).”

The corrected sentence appears below:

“Those individuals whose values on GGT and LDH were below the cut-off point (**Table 4**) presented a better prognosis than the ones who were above; specifically, they presented an OS increase by 39.3%, and 49.6%, respectively (Figures 1B, C). For their part, ALP<102.00 (Figure 1A), number of leukocytes<8,000, neutrophiles<6,000 and monocytes<800 (Figure 1D-F) were also statistically significant, resulting in patients with low levels increasing their survival by 41.8%, 44.9%, 42.4% and 44.3%, respectively (**Table 4**).”

The authors apologize for these errors and state that this does not change the scientific conclusions of the article in any way. The original article has been updated.

